# Fabrication of Polytetrafluoroethylene-Reinforced Fluorocarbon Composite Coatings and Tribological Properties Under Multi-Environment Working Conditions

**DOI:** 10.3390/polym16243595

**Published:** 2024-12-22

**Authors:** Changqing Xi, Bochao Zhang, Xiangdong Ye, Honghua Yan

**Affiliations:** 1 School of Mechanical and Electrical Engineering, Xi’an University of Architecture and Technology, Xi’an 710055, China; xichq@xauat.edu.cn (C.X.); zhang_bch@126.com (B.Z.); yanhh521@xauat.edu.cn (H.Y.); 2 Shaanxi Provincial Key Laboratory of Nanomaterials and Technology, Xian University of Architecture and Technology, Xi’an 710055, China

**Keywords:** polytetrafluoroethylene, fluorocarbon composite coating, orthogonal experiment, multi-environment working conditions, frictional performance

## Abstract

Currently, few studies have been conducted on the use of fluorocarbon resin (FEVE) and polytetrafluoroethylene (PTFE) as adhesive substrates and lubricating and anti-corrosion fillers, respectively, for the fabrication of PTFE-reinforced fluorocarbon composite coatings. In this paper, the tribological properties of polytetrafluoroethylene-reinforced fluorocarbon composite coatings were investigated through orthogonal tests under various operating conditions. The optimal configuration for coating preparation under dry friction and aqueous lubrication was thus obtained: the optimal filler particle size, mass ratio of FEVE to PTFE, spraying pressure, and curing agent content were 50 μm, 3:4.5, 0.3 MPa, and 0.3, respectively. Under oil lubrication, the corresponding optimal values were 5 μm, 3:4.5, 0.3 MPa, and 0.3, respectively. Tribological tests revealed that the best overall performance of the FEVE/PTFE coating was obtained when the mass ratio of FEVE to PTFE was 3:4.5, and the filler particle size also significantly affected the tribological properties under different environments, including the friction coefficients of the FEVE/50 μm-PTFE coating under both dry friction and aqueous lubrication, as well as the friction coefficient of the FEVE/5 μm-PTFE coating under oil lubrication. These coefficients were 0.067, 0.062, and 0.055, representing decreases of 86%, 92%, and 56%, respectively, compared to those of the pure FEVE coating under the same working conditions. This research was conducted with the goal of expanding the application of fluorocarbon coatings in the field of tribology.

## 1. Introduction

Friction between moving parts causes contact surface wear, which seriously affects the normal operation of machinery; thus, mechanical devices are generally protected with coatings to reduce their coefficient of friction and wear and extend their service life [1]. Bonded solid lubrication coating materials are extensively used in high-tech engineering instruments to deal with common tribological problems [2,3,4,5,6,7,8].

**Figure 1:**
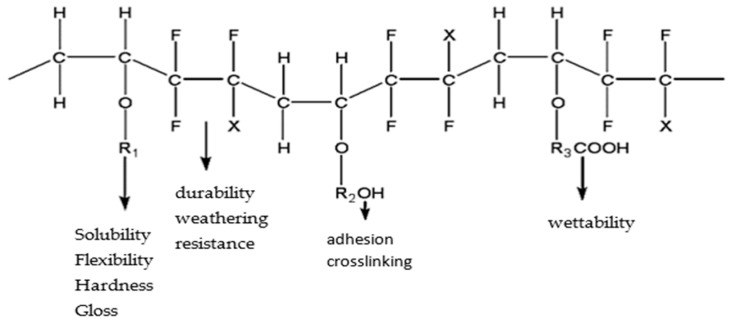
Molecular structure of FEVE.

Bonded solid lubrication coatings typically consist of a binder and a lubricating filler. The curing process of coatings is mainly determined based on the binder type. Although most lubrication coatings are prepared by heat curing, they are not suitable for heat-sensitive substrates because of their high energy consumption during heat curing processes. Fluorocarbon resin (FEVE) is an excellent film-forming material because it can be cured at ambient temperatures and has superb mechanical properties, chemical stability, temperature resistance, and fouling resistance. Figure 1 shows the structural formula of FEVE. Extensive research has been conducted on the tribological properties of fluorocarbon composite coatings. Bodhale A J et al. [9] examined the frictional and wear performances of fluorocarbon (FC)-coated and non-FC-coated HSS M2 parts and noticed that the fluorocarbon coating had exceptional low friction as well as release capability and good chemical properties. As a result of experimentation, fluorocarbon coating shows a coefficient of friction as low as 0.196, which is much better than many other coatings in the fluorocarbon category. Wang et al. [10] prepared pure FC and silica–graphene oxide (SiO2-GO)/FC nanocomposite coatings and observed that the coefficient of friction and wear rate of the composite coating were significantly reduced as compared to those of the pure FC coating after 50 days of the impregnation experiment. Li et al. [11] prepared a series of sodium iron titanate (NFTO)–fluorocarbon composite coatings and systematically investigated the effects of NFTO flakes and NFTO whiskers on the tribological and corrosion behaviors of the composite coatings. The results show that the addition of NFTO can significantly enhance the friction-reducing and wear resistance performances of the fluorocarbon coating. Zhang et al. [12] investigated the effects of the aspect ratio and content of sodium iron titanate NFTO whiskers on the morphology, mechanical properties, and tribological properties of NFTO-reinforced FEVE composite coatings. The results showed that the addition of NFTO whiskers significantly improved the mechanical and wear resistance properties of the composite coatings and reduced the coefficient of friction. Zhou et al. [13] synthesized a fluorinated graphite/fluorocarbon (FGi/FEVE) composite coating by modifying FEVE with FGi. Friction test results show that adding 1% FGi can reduce the friction coefficient of the FEVE coating by 16.9% and the wear rate by 48.0%. Zhou et al. [14] adopted a simple blending method to fabricate an epoxy resin (EP)–modified fluorocarbon resin (FEVE) composite coating (EP/FEVE) and found that when the mass of EP was 10% of the mass of FEVE, the abrasion resistance of the composite coating under dry friction and simulated seawater lubrication conditions increased by 69.8 and 66.1%, respectively. Hence, it can be inferred that fluorocarbon composite coatings have superior friction and corrosion resistance compared to pure fluorocarbon coatings under complex working conditions. Generally, filler modification plays an important role in optimizing the wear-resisting and corrosion-resisting performances of fluorocarbon coatings.

**Figure 2:**
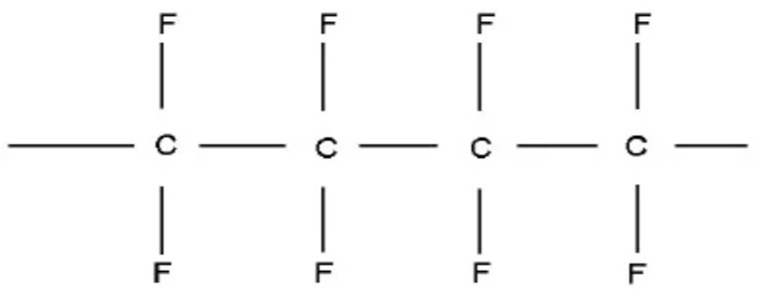
PTFE molecular structure.

Polytetrafluoroethylene (PTFE) is widely used as a self-lubricating material due to its extremely low coefficient of friction [15,16,17,18] and good thermal and chemical stability [15,19,20]. Figure 2 shows the structural formula of FEVE. However, the frictional performance of fluorocarbon resin-bonded solid lubrication coatings with PTFE as a filler (FEVE/PTFE) under multi-environment working conditions has been rarely reported. In the present work, FEVE/PTFE composite coatings were prepared by spraying and room-temperature curing using FEVE as the bonding base material and PTFE as the lubricating and anticorrosive filler, and their tribological properties under multi-environment working conditions were analyzed. The aim of this research is to expand the use of fluorocarbon coatings in tribology and to effectively increase the service life of mechanical moving parts.

## 2. Materials and Methods

### 2.1. Experimental Materials

The reagents used in the experiment and their specifications are shown in Table 1.

The instrumentation to be used in the experiment is detailed in Table 2.

### 2.2. Experimental Design

#### 2.2.1. Preparation of FEVE/PTFE Composite Coatings Based on the Orthogonal Test Principle

The orthogonal experimental design method can effectively reduce the workload of experiments and enhance the convergence speed by selecting a certain proportion of representative points in an orthogonal way. As the preparation of fluorocarbon composite coatings depends on various factors, the present study considered four main factors (PTFE filler particle size, coating composition ratio, gun pressure, and curing agent content) and designed three levels for each factor. Orthogonal tests were designed in the form of four-factor–three-level tests with a total of nine groups of experiments (Table 3).

##### Orthogonal Test Specimen Preparation

The 45 steel substrate of 40 × 40 × 2 mm size was first ground to a surface roughness of 0.8 µm using a grinder, then cleaned with deionized water, soaked in acetone, ultrasonically cleaned for 10 min, and finally, kept in a drying oven.

The spraying solution was configured according to the orthogonal test arrangement presented in Table 3. Firstly, a certain amount of PTFE particles (e.g., 3 g for the preparation of specimen 1) was taken and added to 15 mL of butyl acetate solvent, and ultrasonicated for 10 min to form a homogeneous particle dispersion. Secondly, 3 g of FEVE resin was added to the particle dispersion, and with a rotating speed of 600 r/min, magnetically stirred for 3 h, forming a particle–resin dispersion. Then, a certain amount of curing agent (e.g., 0.3 g for the preparation of specimen 1) was slowly added to the above dispersed liquid system for 30 min of magnetic stirring, to provide the corresponding FEVE/PTFE spray solution. Finally, according to the spraying process configured, the spraying solution was uniformly sprayed on the surface of 45 steel, with a coating thickness of 40 μm, followed by curing at room temperature and forming under 24 h. According to this method, 9 groups of experimental specimens in the orthogonal table were obtained.

##### Orthogonal Experiment

(1) Adhesion test of FEVE/PTFE coatings

Coating adhesion is an important mechanical performance index to assess the degree of adhesion between a coating and its substrate. The adhesion between the FEVE/PTFE coatings and the 45 steel substrate surface was evaluated according to the GB/T 9286-1998 standard (Color Paints and Varnishes—Scratch Test of Coating Films) [21]. The coating surface was cut vertically by a 2 mm cutting knife with uniform force and speed, and five parallel lines with a spacing of 2 mm were drawn in both horizontal and vertical directions to form a regular array of squares. The coating surface was cleaned with a brush, and a 3 M transparent tape was used to make adhesive contact with the coating surface. The tape was quickly torn off after 5 min at an angle of 60°, and the shedding of the coating was visualized to classify the coating adhesion according to the six levels of the grading table.

(2) Tribological performance test of FEVE/PTFE coatings

**Figure 3:**
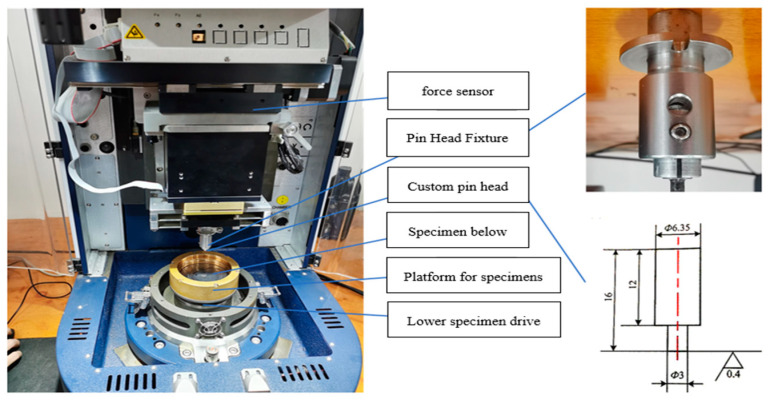
Multifunctional friction and wear tester.

**Figure 4:**
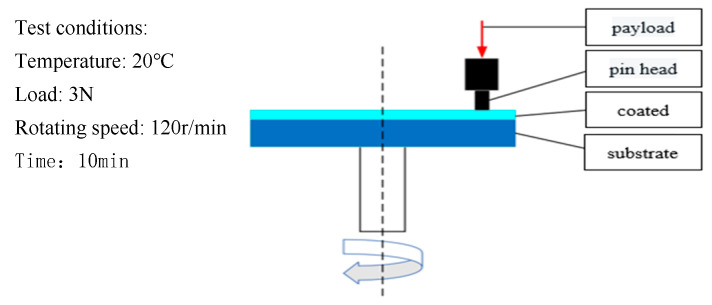
Schematic representation of the friction test principle.

The tribological performance of coatings is an important parameter for evaluating their lubrication characteristics. In this experiment, a UMT-TriboLab testing machine (Figure 3) was used to calculate the coefficient of friction of the FEVE/PTFE coatings under dry friction. The experimental pin-on-disc tribometer had a face-to-face frictional contact form and used a GCr15 pin of 3 mm diameter. All tribological tests were conducted at room temperature (20 °C) under a load of 3 N for 10 min at a rotational speed of 120 rpm with a radius of rotation of 5 mm. The friction test principle is schematically presented in Figure 4.

#### 2.2.2. Frictional Performance Evaluation of FEVE/PTFE Coatings Under Multi-Environment Working Conditions

To examine the frictional performances of the composite coatings selected from the orthogonal tests under multi-environment working conditions, tribological experiments under dry friction and water and oil lubrication were carried out on a multi-functional friction tester. All experiments were performed for 15 min under a load of 3 N at a speed of 120 rpm, and water or oil lubrication was realized by applying 10–15 drops of water or oil per minute to the friction contact surface. A control group was designed with the FEVE to PTFE mass ratios of 3:0 (E0), 3:1.5 (E1), 3:3 (E2), 3:4.5 (E3), and 3:6 (E4) to conduct the frictional performance experiments for PTFE filler particle sizes of 500 nm, 5 μm, and 50 μm. The FEVE to PTFE mass ratios for the spraying solutions are presented in Table 4. The gun pressure and the curing agent parameters were chosen according to the optimized coating formulation parameters.

## 3. Results and Discussion

### 3.1. Optimized Preparation of FEVE/PTFE Composite Coatings

#### 3.1.1. Adhesion Properties of the Composite Coatings

**Figure 5:**
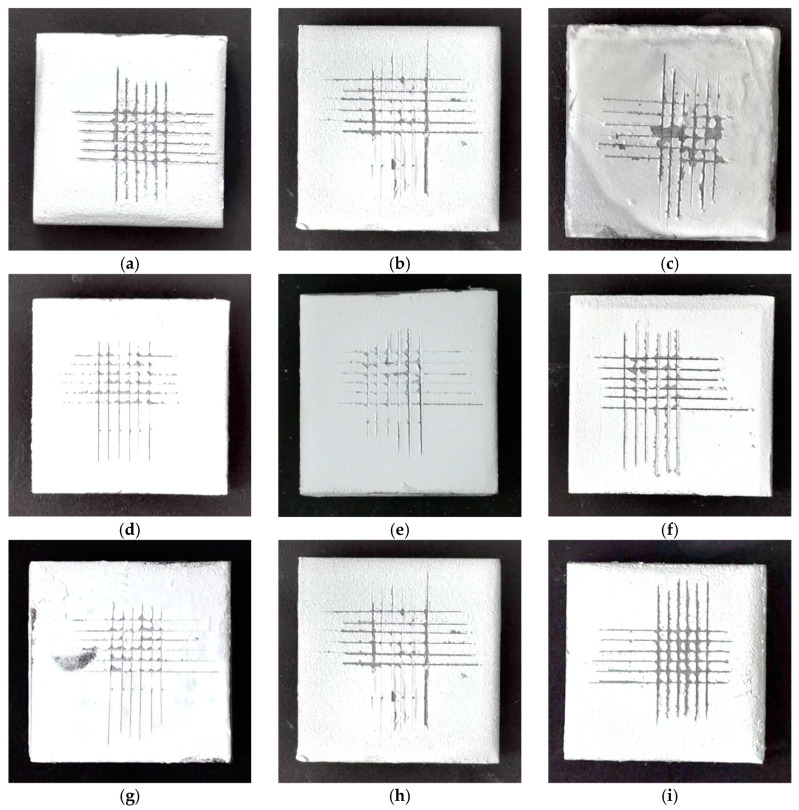
Adhesion topographies of different orthogonal test specimens. (**a**) Specimen no. 1. (**b**) Specimen no. 2. (**c**) Specimen no. 3. (**d**) Specimen no. 4. (**e**) Specimen no. 5. (**f**) Specimen no. 6. (**g**) Specimen no. 7. (**h**) Specimen no. 8. (**i**) Specimen no. 9.

Adhesion level tests were carried out on the coatings prepared according to the parameters of the nine experimental groups presented in Table 1, and photographs were captured to record their surface morphologies after the scribing process (Figure 5).

Significant differences were noticed in the adhesion properties of the coatings prepared with different FEVE/PTFE mass ratios, filler particle sizes, spraying pressures, and curing agent dosages. Specimen 3 had the largest shedding area after the scribing test; thus, the coating exhibited poor adhesion, and its surface was easily damaged even under a small external force. This may be due to the fact that the content of PTFE particles in specimen no. 3 is too high and the resin content is relatively small, resulting in the resin not being able to envelope the filler, which still exists in a granular form, and not able to form a composite coating with a good bonding force. Coatings 1, 2, 6, and 9 had a large shedding area after the scribing test, indicating poor adhesion between the resin and the filler; therefore, their surfaces were easily damaged under the action of a large external force. Coatings 4, 5, 7, and 8 had a small shedding area after the scribing test, indicating good bonding between the coatings and the substrate.

#### 3.1.2. Tribological Properties of the Composite Coatings

**Figure 6:**
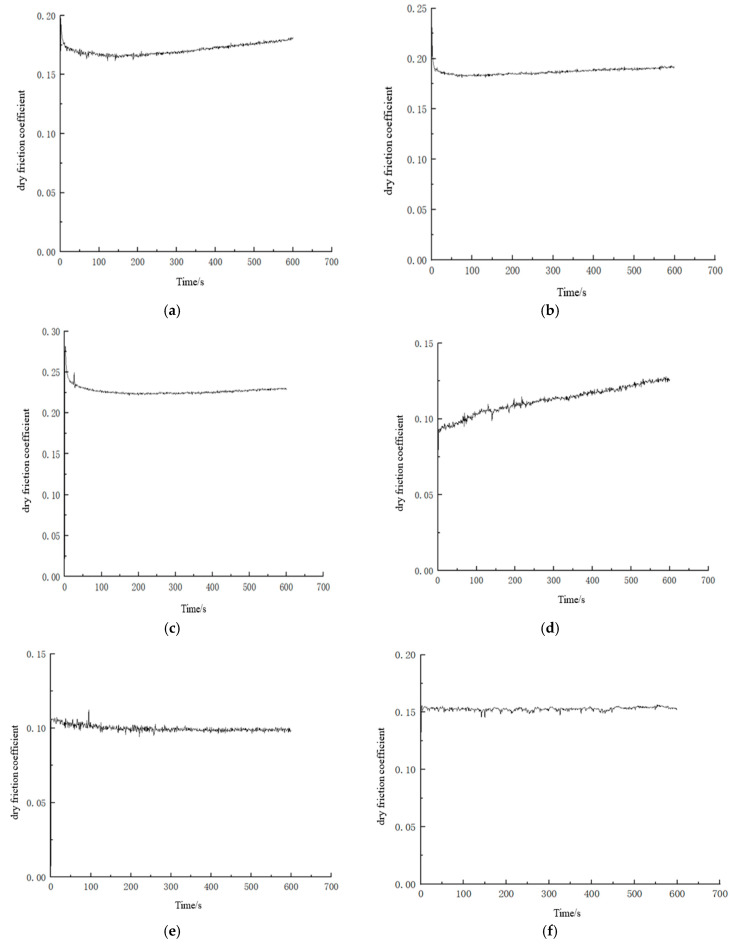

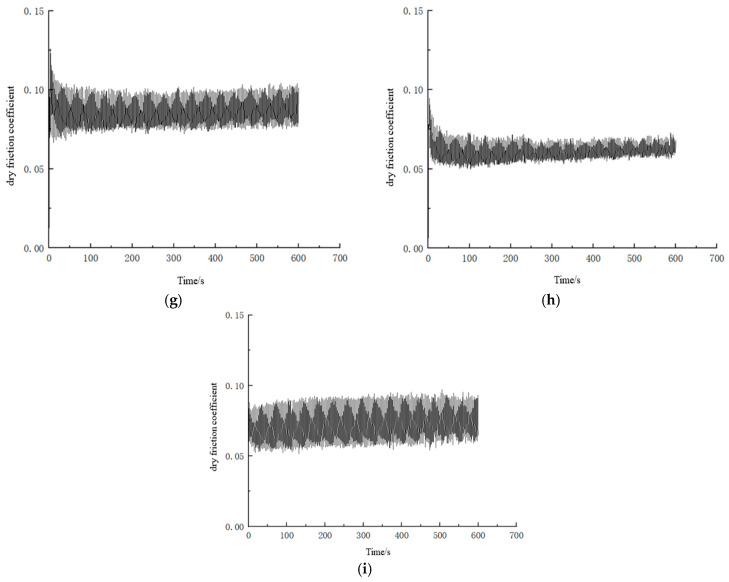
Variations in the coefficient of friction curves of different orthogonal test specimens. (**a**) Specimen no. 1. (**b**) Specimen no. 2. (**c**) Specimen no. 3. (**d**) Specimen no. 4. (**e**) Specimen no. 5. (**f**) Specimen no. 6. (**g**) Specimen no. 7. (**h**) Specimen no. 8. (**i**) Specimen no. 9.

Tribological tests were carried out on the coatings prepared according to the parameters of the nine experimental groups presented in Table 1, and the variations in their coefficient of friction curves are displayed in Figure 6. Each group was tested three times and their average value is the coefficient of friction ($\bar{\mu }$) for that group (see Table 5).

It is discernible from Figure 6 that the fluctuations in the coefficient of friction curves of the composite coatings with PTFE particle sizes of 0.5 μm and 5 μm were smaller than those for the coatings with a PTFE particle size of 50 μm. This is mainly because of the fact that under large particle size conditions, the coating has a larger surface bearing capacity and more lubricating filler after curing and forming, but the surface of the coating specimen formed by the spraying process has a larger roughness, thus resulting in a smaller friction coefficient but a larger fluctuation of the coefficient of friction. In addition, the large particle size filler was also able to attenuate the fluctuation of the coefficient of friction of the coating at a formulation ratio of 3:4.5. The main reason was that the components could be uniformly dispersed at this coating formulation ratio, which resulted in a better leveling of the coating system after curing at room temperature.

The composite coating with a PTFE particle size of 0.5 μm had larger friction coefficients (0.167, 0.168, and 0.231 at the FEVE/PTFE mass ratios of 3:3, 3:4.5, and 3:6, respectively) and experienced very low fluctuations in the coefficient of friction curves (Figure 6a–c). The composite coating with a PTFE particle size of 50 μm had smaller friction coefficients (0.083, 0.067, and 0.075 at the FEVE/PTFE mass ratios of 3:3, 3:4.5, and 3:6, respectively) and experienced large fluctuations in the coefficient of friction curves (Figure 6g–i). The friction coefficients of the coating with a PTFE particle size of 5 μm (0.112, 0.131, and 0.158 at the FEVE/PTFE mass ratios of 3:3, 3:4.5, and 3:6, respectively) were in between those of the coatings with PTFE particle sizes of 0.5 and 50 μm, and their coefficient of friction curve fluctuations also followed the same trend; thus, they yielded better overall performance. The coating with a PTFE particle size of 5 μm had a better bonding force at all FEVE/PTFE mass ratios, and PTFE wafers generated by friction experienced relative sliding between the contact surfaces and in turn, reduced the coefficient of friction; thus, the coating had a certain degree of stability.

PTFE microparticles continuously formed an effective transfer lubrication film between the friction contact surfaces, whereas as PTFE nanoparticles had poor carrying capacity, the lubrication film fell off and abrasive debris was formed with the rise in friction time; thus, the friction resistance increased, making the coefficient of friction larger. When the FEVE/PTFE mass ratio was 3:6, the presence of a large amount of filler in the coating led to a smaller bonding force and a larger coefficient of friction.

#### 3.1.3. Analysis of Orthogonal Test Results

The extreme difference method is most commonly used in orthogonal analysis. In this technique, the extreme difference (R) refers to the difference between the maximum and minimum values of the average value of the test indicators corresponding to each level in each column. The average value of the summation of the level indicators for each factor (k-avg) is calculated through the summation of the level indicators of each factor (K), and the value of R is obtained based on k-avg (R reflects the advantages and disadvantages of each factor, whereas k-avg indicates the level of advantages and disadvantages). The extreme difference analysis results for the adhesion levels and friction coefficients of the nine groups of orthogonal test specimens are listed in Table 5.

(1) Selection of the optimal combination of factors affecting the adhesion grade

**Figure 7:**
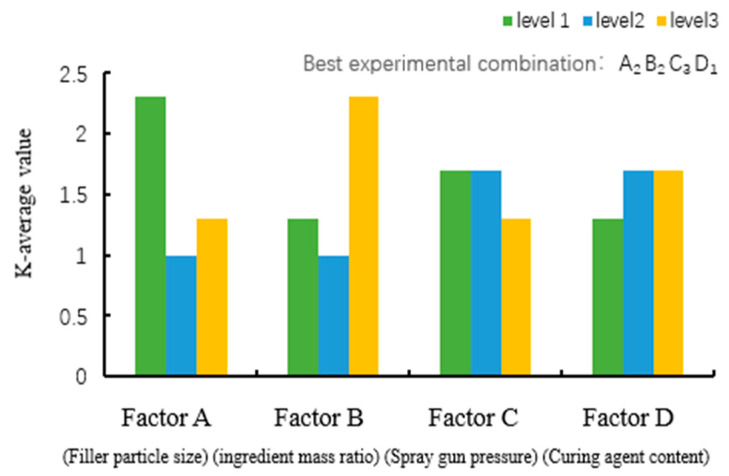
Plot of the mean values of levels for adhesion factors.

Figure 7 plots the average values of levels for adhesion factors. The order of factors affecting the adhesion grade was RA = RB > RC = RD, where A is the filler particle size, B is the FEVE/PTFE mass ratio, C is the spraying gun pressure, and D is the curing agent content. The extreme differences for factors A and B were 1.3, indicating that the filler particle size and the FEVE/PTFE mass ratio had the greatest influence on the adhesion grade, whereas the extreme differences for factors C and D were 0.4, implying that the gun pressure and the curing agent content had a smaller impact.

The order of levels for factor A affecting the adhesion grade was A1 > A3 > A2. It is evident that the coating had the best adhesion strength for the PTFE particle size of 5 μm, indicating that the PTFE particle size could not be too large or too small to obtain the best adhesion force; thus, the optimal level of factor A was selected as A2. The order of levels for factor B affecting the adhesion grade was B3 > B1 > B2. It is observable that the amount of PTFE particles could not be too high or too little to achieve the highest adhesion strength; thus, the optimal level of factor B was selected as B2 (FEVE: PTFE = 3:4.5). The order of levels for factor C affecting the adhesion grade was C1 = C2 > C3; indicating that the spraying gun pressure of 0.3 MPa resulted in the largest adhesion force; thus, the optimal level of factor C was selected as C3. The order of levels for factor D affecting the adhesion grade was D1 < D2 = D3, revealing that the curing agent content of 0.3 g yielded the highest adhesion strength; hence, the optimal level of factor D was chosen as D1. Therefore, the optimal combination to achieve the best adhesion grade was A2B2C3D1.

(2) Selection of the optimal combination of factors affecting the friction coefficient

**Figure 8:**
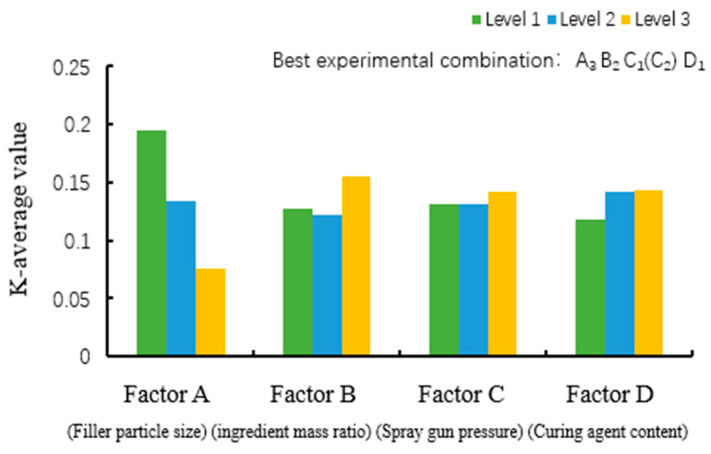
Plot of the mean values of levels for friction factors.

Figure 8 plots the average values of levels for friction factors. The order of factors affecting the friction factor was determined as RA > RB > RD > RC. The extreme difference of factor A (0.12) was larger than those of the other factors, indicating that the filler particle size had the greatest influence on the friction coefficient. The extreme difference of factor C was found to be the smallest (0.011); thus, the gun pressure had the smallest influence.

The order of levels for factor A affecting the friction factor was A1 > A2 > A3, implying that the coating had the lowest coefficient of friction when the PTFE particle size was 5 μm; thus, the optimal level of factor A was chosen as A3. The order of levels for factor B affecting the friction factor was B3 > B1 > B2. It is noticeable that the coating had the lowest friction coefficient for the FEVE/PTFE ratio of 3:4.5; therefore, the optimal level of factor B was chosen as B2. The order of levels for factor C affecting the friction factor was C1 = C2 < C3. It is clear that the friction coefficient was the smallest when the spraying gun pressure was 0.3 MPa; thus, the optimal level of factor C was selected as C1(C2). The order of levels for factor D affecting the friction factor was D3 > D2 > D1, implying that the curing agent content of 0.3 g caused the lowest friction coefficient; hence, the optimal level of factor D was selected as D1. Therefore, the optimal combination to obtain the lowest coefficient of friction was A3B2C1 (C2) D1.

(3) Determination of the optimal factor level combination

Under dry friction and water lubrication, as the PTFE filler particle size increased, PTFE particles started to accumulate on the coating surface and formed a continuous and dense wetting transfer film, which promoted coating friction reduction and lubrication; thus, the optimal level for factor A was chosen as A3.

Under oil lubrication, the lubrication state was better than that under dry friction and water lubrication, and the optimal level for factor A was selected as A2 because this state had the lowest influence on the adhesion and friction factor. Similarly, the optimal levels for factors B, C, and D were selected as B2, C2, and D1, respectively, because these states caused the largest adhesion strength and the lowest friction coefficient.

Therefore, under dry friction and water lubrication, the optimal combination for coating preparation was A3B2C2D1 (PTFE filler particle size = 50 μm, FEVE/PTFE mass ratio = 3:4.5, spraying gun pressure = 0.25 MPa, and curing agent content = 0.3 g), and under oil lubrication, the optimal combination was A2B2C2D1 (PTFE filler particle size = 5 μm, FEVE/PTFE mass ratio = 3:4.5, spraying gun pressure = 0.25 MPa, and curing agent content = 0.3 g).

### 3.2. Frictional Properties of FEVE/PTFE Composite Coatings Under Multi-Environment Working Conditions

#### 3.2.1. Tribological Properties Under Dry Friction

**Figure 9:**
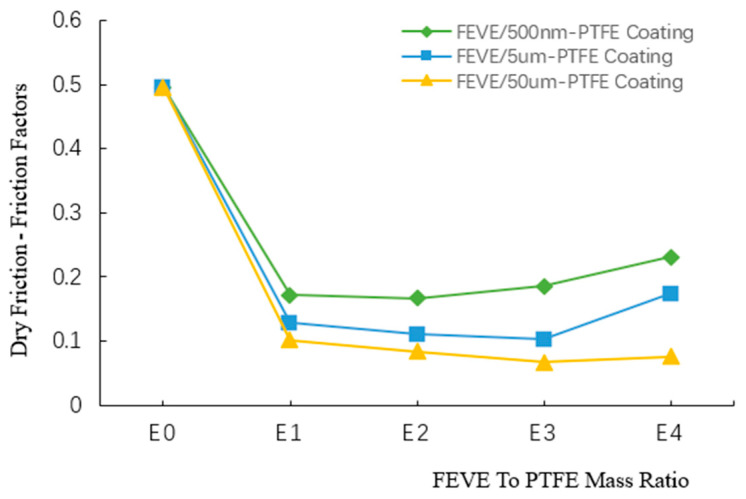
Friction coefficient variation curves of the composite coatings with different FEVE/PTFE mass ratios under dry friction.

The friction coefficient variation curves of the composite coatings with different FEVE/PTFE mass ratios under dry friction are exhibited in Figure 9 (Specific values are given in Schedule S1 of the Supplementary Material). When the FEVE/PTFE mass ratio was E0, the friction coefficient of the pure resin coatings was about 0.4959. The friction coefficients of all coatings decreased rapidly at the FEVE/PTFE mass ratio of E1. The friction coefficients of the coatings first decreased and then gradually increased as the FEVE/PTFE mass ratio was raised from E1 to E4. The FEVE/500 nm-PTFE coating had the lowest friction coefficient of about 0.167 at the FEVE/PTFE mass ratio of E2. The FEVE/5 μm-PTFE coating and the FEVE/50 μm-PTFE coating had the lowest friction coefficients of 0.103 and 0.067, which were reduced by 79 and 86% compared to that of the pure FEVE coating, respectively, at the FEVE/PTFE mass ratio of E3. Molecular chains of PTFE possess a helical structure and yield a lubricating effect; hence, when PTFE was added to FEVE in an appropriate amount to improve the densification of the coating, the surface load-bearing capacity of the coating was enhanced. However, when the FEVE/PTFE mass ratio was too large, the coating was prone to cracking and peeling off, causing an increase in the coefficient of friction.

The FEVE/500 nm-PTFE coating had the largest friction coefficient, followed by the FEVE/5 μm-PTFE and FEVE/50 μm-PTFE coatings; thus, the FEVE/50 μm-PTFE coating manifested a better self-lubricating effect. The PTFE filler with a 500 nm particle size had a large surface area, and its bonding with the FEVE resin was stronger, inhibiting the formation of a transfer film on the coating and resulting in a larger coefficient of friction. When the particle size of PTFE was raised to 5 or 50 μm, PTFE particles accumulated on the coating surface and formed a continuous and dense lubrication transfer film, which promoted coating friction reduction and lubrication. Therefore, the FEVE/50 μm-PTFE coating had excellent friction reduction properties under dry friction at the FEVE/PTFE mass ratio of E3, verifying the effectiveness of the A3B2C2D1 formulation under dry friction.

#### 3.2.2. Tribological Properties Under Water Lubrication

**Figure 10:**
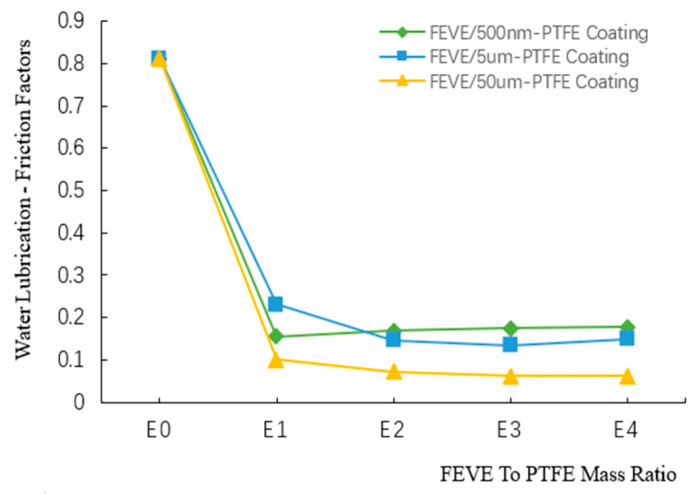
Friction coefficient variation curves of the composite coatings with different FEVE/PTFE mass ratios under water lubrication.

The friction coefficient variation curves of the composite coatings with different FEVE/PTFE mass ratios under water lubrication are presented in Figure 10 (Specific values are given in Schedule S2 of the Supplementary Material). When the FEVE/PTFE mass ratio was E0, the friction coefficient of the FEVE/PTFE coatings was about 0.813. When the FEVE/PTFE mass ratio was E1, the friction coefficients of all coatings were reduced rapidly. When the FEVE/PTFE mass ratio was raised from E1 to E4, the friction coefficient of the FEVE/500 nm-PTFE coating gradually increased and reached 0.178 at E4. When the FEVE/PTFE mass ratio was raised from E1 to E4, the friction coefficients of the FEVE/5 μm-PTFE and FEVE/50 μm-PTFE coatings first decreased and then gradually increased. The FEVE/5 μm-PTFE and FEVE/50 μm-PTFE coatings had the minimum friction coefficients of 0.134 and 0.062 (84 and 92% lower than that of the pure FEVE coating), respectively, at the FEVE/PTFE mass ratio of E3.

Unlike the friction coefficient curves under dry friction, the friction coefficient curves of the coatings under water lubrication continuously decreased. This occurred because of the water boundary lubrication layer between the coating surface and the friction specimen with a certain lubricating effect, but this was not favorable for the coatings due to the low viscosity of water. The friction coefficient of the FEVE/5 μm-PTFE coating under water lubrication increased because water molecules penetrated the coating surface and reduced the shear strength of the coating, causing an increase in the friction coefficient. Hence, the FEVE/50 μm-PTFE coating had good friction reduction performance under water lubrication at the FEVE/PTFE mass ratio of E3, verifying the effectiveness of the A3B2C2D1 formulation under water lubrication.

#### 3.2.3. Tribological Properties Under Oil Lubrication

**Figure 11:**
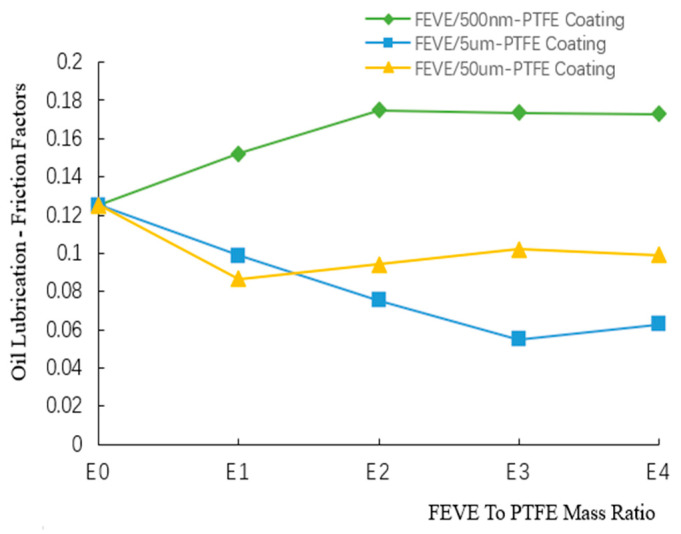
Friction coefficient variation curves of the composite coatings with different FEVE/PTFE mass ratios under oil lubrication.

The friction coefficient variation curves of the composite coatings with different FEVE/PTFE mass ratios under oil lubrication are illustrated in Figure 11 (Specific values are given in Schedule S3 of the Supplementary Material). When the FEVE/PTFE mass ratio was E0, the friction coefficient of the coatings was 0.125. The friction coefficients of the FEVE/5 μm-PTFE and FEVE/50 μm-PTFE coatings decreased at the FEVE/PTFE mass ratio of E1, whereas the friction coefficient of the FEVE/500 nm-PTFE coating increased. When the FEVE/PTFE mass ratio was raised from E1 to E4, the friction coefficient of the FEVE/500 nm-PTFE coating gradually increased and reached 0.173 at E3, whereas the friction coefficient of the FEVE/5 μm-PTFE coating first decreased to a minimum of 0.055 at E3 (56% lower than that of the pure FEVE coating) and then gradually increased. Similarly, the coefficient of friction of the FEVE/50 μm-PTFE coating gradually increased and reached about 0.1 at E3.

The FEVE/500 nm-PTFE coating had the largest friction coefficient, followed by the FEVE/50 μm-PTFE and the FEVE/5 μm-PTFE coatings. The hardness of the FEVE/500 nm-PTFE coating was low, and suspended PTFE particles generated during friction easily moved to the contact surface and inhibited the generation of a PTFE transfer film in the oil-lubricated contact area; thus, the FEVE/500 nm-PTFE coating had poor friction reduction performance. The FEVE/5 μm-PTFE coating manifested good friction reduction performance under oil lubrication at the FEVE/PTFE mass ratio of E3, verifying the effectiveness of the A2B2C2D1 formulation under oil lubrication.

#### 3.2.4. Discussion on the Tribological Performance of the Composite Coatings Under Multi-Environment Working Conditions

It was found that the tribological performance of the composite coatings was greatly affected by the mass ratio of the binder and the lubricating filler, because PTFE acted as a functional filler with a lubricating effect; thus, when it was abraded against hard materials, abrasive debris formed by wafer fracture formed a transfer film in the sliding direction. The presence of the transfer film transformed the friction between the coating and the substrate into a frictional contact and reduced the coefficient of friction. When the percentage of PTFE in the coating formulation was low, it was difficult to generate an effective lubrication transfer film on the coating surface; hence, the friction reduction effect of the coating was significantly affected. Therefore, when the proportion of PTFE was E3, the friction produced a complete transfer film that contributed to the reduction of the coefficient of friction. Therefore, the overall performance of the coating was better at the FEVE/PTFE mass ratio of E3.

**Figure 12:**
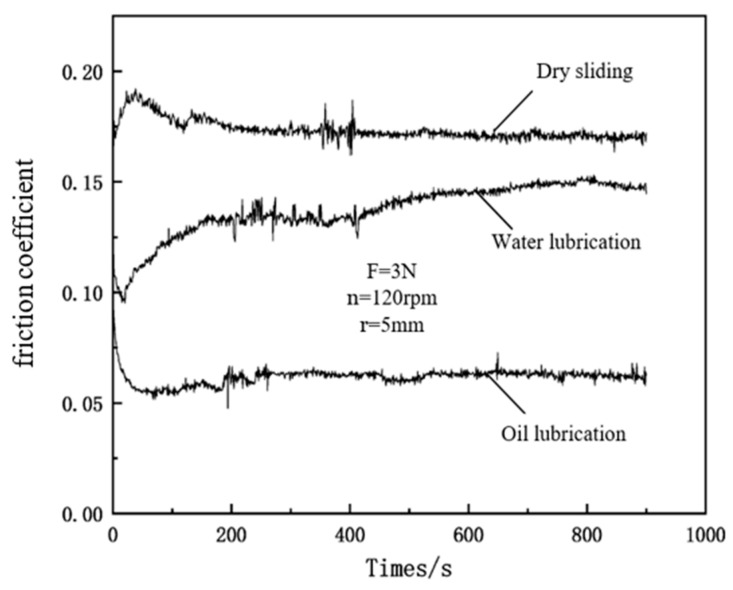
Friction coefficient variation curves of the FEVE/5 μm-PTFE composite coating under different working conditions.

Figure 12 displays the friction coefficient variation curves of the FEVE/5 μm-PTFE composite coating under different working environments. The friction coefficients of the FEVE/5 μm-PTFE coating under dry friction and water and oil lubrication were determined as 0.173, 0.149, and 0.063, respectively, at the FEVE/PTFE mass ratio of E4. The liquid lubrication reduced the friction coefficient of the coating, and the lubrication effect of oil was better than that of water given that the viscosity of oil is much higher, thus generating a boundary lubrication layer with a larger thickness and further reducing the coefficient of friction with a better boundary lubrication effect. Experiments have shown that the availability of solid lubricant coatings together with liquid lubricants can further optimize the friction performance.

## 4. Conclusions

In this study, PTFE-reinforced fluorocarbon composite coatings were fabricated by spraying and room-temperature curing, and their tribological properties under multi-environment working conditions were systematically investigated based on orthogonal tests. The key findings of this work are delineated below.

(1) The FEVE/PTFE coating formulation was optimized for orthogonal experiments. The filler particle size, the FEVE/PTFE mass ratio, the spraying pressure, and the curing agent content were selected as the four main factors, and three levels were designed for each factor. Under dry friction and water lubrication, the best experimental combination was determined as A3B2C2D1 (PTFE filler particle size = 50 μm, FEVE/PTFE mass ratio = 3:4.5, spraying pressure = 0.25 MPa, and curing agent content = 0.3 g), whereas under oil lubrication, the optimal combination was found to be A2B2C2D1 (PTFE filler particle size = 5 μm, FEVE/PTFE mass ratio = 3:4.5, spraying pressure = 0.25 MPa, and curing agent content = 0.3 g).

(2) The frictional properties of the optimized FEVE/PTFE composite coatings were investigated under multi-environment working conditions, and it was noticed that the PTFE content and the lubrication condition had a significant effect on the tribological properties of the coatings. When the FEVE/PTFE mass ratio was E3 (3:4.5), the friction coefficients of the FEVE/50 μm-PTFE coating under dry friction and water lubrication were 0.067 and 0.062, which were 86 and 92% lower than those of the pure FEVE coating, respectively. The friction coefficient of the FEVE/5 μm-PTFE coatings in oil lubrication was 0.055, which was 56% lower than that of the pure FEVE coating. It can thus be seen that the FEVE/PTFE coating can further reduce the coefficient of friction, and at the same time, selecting lubricating fillers with better system compatibility can effectively reduce this friction coefficient.

(3) The tribological properties of the FEVE/PTFE composite coatings manifested a strong relationship with the mass ratio of the binder and the lubricating filler. When the PTFE content in the coating formulation was low, it was difficult to obtain an effective lubrication transfer film on the coating surface; thus, the friction reduction effect of the coating was greatly affected. When the proportion of PTFE reached E3, the friction produced a complete transfer film on the coating surface, prompting a reduction in the friction coefficient. The best overall performance of the FEVE/PTFE coating was obtained when the mass ratio of FEVE to PTFE was E3.

## Figures and Tables

**Table 1:** Reagent specifications and origins.

Material Name	Model/Specification	Manufacturer
FEVE Fluorocarbon Resin (JF-2X)	industrial grade	Shanghai East Fluorine Chemical Technology Co., Shanghai, China
Polytetrafluoroethylene (500 nm, 5 μm, and 50 μm)	industrial grade	Suzhou Huihuang Fluorine Plastic Chemical Co., Suzhou, China
Bayer 3390 Curing Agent	industrial grade	Jining Huakai Resin Co., Jining, China
Nanosilicon dioxide (500 nm)	industrial grade	Degussa Chemical Co., Qingdao, China
Butyl acetate	industrial grade	Xi’an Meiju Trade Co., Xi’an, China
Ethanol	industrial grade	National Group Chemical Reagent Co., Beijing, China
Concentrated sulfuric acid H_2_SO_4_	industrial grade	National Group Chemical Reagent Co., Beijing, China
Acetone (CH_3_COCH_3_)	industrial grade	National Group Chemical Reagent Co., Beijing, China
Deionized water	-	Configured ourselves

**Table 2:** Specification models and manufacturers of experimental instruments.

Instrument Name	Model/Specification	Manufacturer
Electronic balance	YH-A 6002	Rui’an Yingheng Electric Appliance Co., Rui’an, China
Magnetic Stirrer	524G	Shanghai Meiyinpu Instrument Manufacturing Co., Shanghai, China
Spray gun	W-71	Wenli Qidong Co., Qidong, China
Ultrasonic vibrator	500W	Hangzhou Farrant Ultrasonic Technology Co., Hangzhou, China
Thickness gauge	Minitest 70F	EPK Germany, Saarbrücken, Germany
Optical microscope	BX53M	Olympus Corporation, Tokyo, Japan
Multi-functional friction and wear tester	UMT-TriboLab	Brooke Group USA, Great Neck, NY, USA
Micro scanning electron microscope	S-3000	Hitachi Group Japan, Tokyo, Japan

**Table 3:** Table of orthogonal experiments.

Test Number	Filler Particle Size (μm)	FEVE/PTFE Mass Ratio (FEVE/g:PTFE/g)	Spraying Gun Pressure (MPa)	Curing Agent Content (g)
1	1 (0.5)	1 (3:3)	1 (0.2)	1 (0.3)
2	1 (0.5)	2 (3:4.5)	2 (0.25)	2 (0.5)
3	1 (0.5)	3 (3:6)	3 (0.3)	3 (0.7)
4	2 (5)	1 (3:3)	2 (0.25)	3 (0.7)
5	2 (5)	2 (3:4.5)	3 (0.3)	1 (0.3)
6	2 (5)	3 (3:6)	1 (0.2)	2 (0.5)
7	3 (50)	1 (3:3)	3 (0.3)	2 (0.5)
8	3 (50)	2 (3:4.5)	1 (0.2)	3 (0.7)
9	3 (50)	3 (3:6)	2 (0.25)	1 (0.3)

**Table 4:** FEVE/PTFE mass ratio in one spray solution.

Mass Ratio	E0 (3:0)	E1 (3:1.5)	E2 (3:3)	E3 (3:4.5)	E4 (3:6)
FEVE	3 g	3 g	3 g	3 g	3 g
PTFE	0 g	1.5 g	3 g	4.5 g	6 g

**Table 5:** Extreme variance analysis results.

Experiment Number		Experimental Factors				Experimental Results	
		Filler Particle Size (A)	FEVE/PTFE Mass Ratio (B)	Spray Gun Pressure (C)	Curing Agent Content (D)	Adhesion (Grade)	Friction Coefficient ($\bar{\mu }$)
1		1	1	1	1	2	0.167
2		1	2	2	2	2	0.186
3		1	3	3	3	3	0.231
4		2	1	2	3	1	0.131
5		2	2	3	1	0	0.112
6		2	3	1	2	2	0.158
7		3	1	3	2	1	0.083
8		3	2	1	3	1	0.067
9		3	3	2	1	2	0.075
Adhesion level	K1	7	4	5	4	Summation of each adhesion level indicator	
	K2	3	3	5	5		
	K3	4	7	4	5		
	k1	2.3	1.3	1.7	1.3	Mean value of the sum of each adhesion level indicator	
	k2	1	1	1.7	1.7		
	k3	1.3	2.3	1.3	1.7		
	R	1.3	1.3	0.4	0.4		
Optimal solution		A2	B2	C3	D1		
Friction factor	K1	0.584	0.381	0.392	0.354	Summation of the level indicators for each friction coefficient	
	K2	0.401	0.365	0.392	0.427		
	K3	0.225	0.464	0.426	0.429		
	k1	0.195	0.127	0.131	0.118	Average of the sum of each friction coefficient level indicator	
	k2	0.134	0.122	0.131	0.142		
	k3	0.075	0.155	0.142	0.143		
	R	0.12	0.033	0.011	0.025		
Optimal solution		A3	B2	C1(C2)	D1		

## Data Availability

Data are contained within the article or Supplementary Material.

## Supplementary Materials

The following supporting information can be downloaded at: https://www.mdpi.com/article/10.3390/polym16243595/s1, Schedule S1: Test results obtained under dry friction conditions; Schedule S2: Test results under water lubrication conditions; Schedule S3: Test results under oil lubrication condition
