# Supplementary material for: Fabrication of Polytetrafluoroethylene-Reinforced Fluorocarbon Composite Coatings and Tribological Properties Under Multi-Environment Working Conditions
Source: Polymers (Basel). 2024 Dec 22;16(24):3595. doi: 10.3390/polym16243595 (PMC11678996; doi:10.3390/polym16243595)
Supplement: Supplementary file 1 [file polymers-16-03595-s001.zip › polymers-3340520-supplementary.pdf]

Supplementary File

Schedule 1 Test results obtained under dry friction conditions

PTFE particle size	E0	E1	E2	E3	E4
500nm	0.4959	0.1717	0.1671	0.1858	0.2311
5μm	0.4959	0.1278	0.1106	0.1028	0.1732
50μm	0.4959	0.102	0.08344	0.06717	0.07538

Schedule 2: Test results under water lubrication conditions

PTFE particle size	E0	E1	E2	E3	E4
500nm	0.8128	0.1545	0.168	0.1741	0.1781
5μm	0.8128	0.2315	0.1467	0.1338	0.1488
50μm	0.8128	0.1008	0.0717	0.06153	0.06213

Schedule 3 Test results under oil lubrication condition

PTFE particle size	E0	E1	E2	E3	E4
500nm	0.1252	0.1521	0.1749	0.1737	0.1729
5μm	0.1252	0.0988	0.0753	0.055	0.0628
50μm	0.1252	0.08642	0.09428	0.1022	0.0992

CERTIFICATE

OF ENGLISH LANGUAGE EDITING

Research on the fabrication of polytetrafluoroethylene-reinforced fluorocarbon composite coatings and tribological properties under multi-environment working conditions

Currently, few studies have been conducted on the use of fluorocarbon resin (FEVE) and polytetrafluoroethylene (PTFE) as adhesive substrates and lubricating and anti-corrosion fillers, respectively, for the fabrication of PTFE-reinforced fluorocarbon composite coatings. In this paper, the tribological properties of polytetrafluoroethylene-reinforced fluorocarbon composite coatings were investigated through orthogonal tests under various operating conditions. The optimal configuration for coating preparation under dry friction and aqueous lubrication was thus obtained: the optimal filler particle size, mass ratio of FEVE to PTFE, spraying pressure, and curing agent content were 50 μm, 3:4.5, 0.3 MPa, and 0.3, respectively. Under oil lubrication, the corresponding optimal values were 5 μm, 3:4.5, 0.3 MPa, and 0.3, respectively. Tribological tests revealed that the best overall performance of the ...

This document certifies that the manuscript listed above was copy edited for English language by LetPub, with regard to grammar, punctuation, spelling, and clarity. Documents receiving this certification should be regarded as having undergone professional editorial revision for English language before submission. However, the authors may accept or reject LetPub's suggestions and changes at their own discretion and LetPub does not have editorial control over the submitted documents. Submitted documents may have new text that was not provided to LetPub for review. Please use the verification link below to determine the validity of the submitted version.

December 10, 2024

Date of Revision

LetPub

LetPub is an author service brand owned and operated by Accdon LLC.
Tel: 1-781-202-9968 Email: info@accdon.com
Address: 400 Fifth Ave. Suite 530, Waltham, MA 02451, United States

This manuscript has been individually edited for grammar, punctuation, spelling, and clarity. You may verify the authenticity of this certificate on our website (https://www.letpub.com/editorial-certificate) at any time using this manuscript's unique code: PR_241004Z840Y v241209.

© 2024 Accdon, LLC.
